# Detection of O6-methylguanine-DNA methyltransferase gene methylation status in IDH-wild type glioblastomas using methylation-specific qPCR: a first report from Morocco

**DOI:** 10.11604/pamj.2024.49.110.45250

**Published:** 2024-12-06

**Authors:** Anass Oukhdouch, Abdelmalek Hakmaoui, Basma Zinbi, Souad Sellami, Bouighajd Hayat, Hanane Rais

**Affiliations:** 1 Laboratory of Biopathology, Center of Clinical Research, Anatomic Pathology Department, Mohammed VI University Hospital Center, Marrakech, Morocco,; 2 Morphoscience Research Laboratory, Faculty of Medicine and Pharmacy, Cadi Ayyad University, Marrakech, Morocco,; 3 Laboratory of Biopathology, Center of Clinical Research, Mohammed VI University Hospital Center, Marrakech, Morocco,; 4 Immunohistochemistry, Anatomic Pathology Department, Mohammed VI University Hospital Center, Faculty of Medicine and Pharmacy, Cadi Ayyad University, Marrakech, Morocco,; 5 Neurosurgery Department, Mohammed VI University Hospital Center, Cadi Ayyad University, Marrakech, Morocco,; 6 Laboratory of Anthropogenic, Biotechnology and Health, Nutritional Physiopathology, Neurosciences and Toxicology Team, Faculty of Sciences, Chouaib Doukkali University, El Jadida, Morocco

**Keywords:** MGMT methylation, immunohistochemistry, MS-qPCR- glioblastoma

## Abstract

We tried, through this work, to highlight the first detection of the methylation status of the O6-methylguanine-DNA methyltransferase gene (MGMT) promoter in IDH-wild-type glioblastomas with observation of the expression of IDH1, ATRX, and P53 by immunohistochemistry. Across eight formalin-fixed, paraffin-embedded tissue blocks (FFPE) from glioblastoma patients, we collected tumor tissue samples. We examined the methylation status of the MGMT gene promoter using a real-time methylation-specific PCR technique (MS-qPCR). In addition, we observed the molecular alterations caused by mutations in IDH1, ATRX, and p53 using immunohistochemistry (IHC). All cases studied had a wild-type form of IDH1, loss of ATRX expression was observed in five samples. In two cases, the mutated form of p53 was expressed. The level of p53 expression ranged from intense (>80% of tumor cells) to weak (>10% of tumor cells), and less than 10% labeling was considered negative. In our study, when analyzing the methylation profiles of the MGMT gene promoter, we found that two cases, specifically sample 2 and sample 5, exhibited positive methylation. Sample 2 had a methylation rate of 6.8%, while sample 5 showed a methylation rate of 27%. In contrast, sample 6 had a methylation rate of 0.05%, which fell below the established methylation cutoff point of 0.6%. Therefore, sample 6 was considered negatively methylated because its methylation rate was significantly lower than the established threshold. This study identifies MGMT promoter methylation in a subset of glioblastomas IDH-wild-type. These findings highlight the molecular diversity of glioblastomas and suggest potential targets for tailored therapeutic strategies.

## Introduction

Gliomas are the most common type of primary malignant brain tumors in adults and are categorized by the World Health Organization (WHO) into four grades (1 to 4), depending on how aggressive they are, glioblastomas are considered to be the most malignant and aggressive gliomas [1]. Glioblastoma is an infiltrating tumor proliferation of high-density astrocytic or indeterminate phenotype [2].

GBMs may exhibit aberrant expression of various growth-control genes and their corresponding proteins, including mutant IDH1 (R132H) expression (IDH1+), loss of alpha thalassemia/mental retardation syndrome X-linked (ATRX) expression (ATRX-) and p53 overexpression (p53+) [3]. GBM is divided into two distinct categories: Primary GBM constitutes about 90% of cases and develops spontaneously [4]. It primarily affects elderly patients and is characterized by genetic alterations such as EGFR gene amplification, EGFR protein overexpression, and loss of the tumor suppressor gene phosphatase and tensin homolog (PTEN) [4]. Secondary GBM, comprising approximately 10% of cases, occurs in younger patients and originates from lower-grade precursor lesions. Secondary GBM generally has a better prognosis and is associated with mutations in the IDH1 and TP53 genes [4].

The current standard treatment approach for newly diagnosed malignant glioma patients involves performing the maximum safe resection followed by a combination of radiotherapy and chemotherapy, mostly with temozolomide (TMZ) [5]. Several parameters are used to predict the theragnostic and orientation of treatment (efficacity of TMZ) of patients with glioblastoma, including epigenetic modification of the O6-methylguanine-DNA methyltransferase (MGMT) gene [6]. Temozolomide is an alkylating agent that induces apoptosis by imparting a methyl group to DNA purine bases principally in O6-guanine, is an alkylazine cytotoxic drug. However, MGMT, a DNA repair protein, reverses alkylation at the O6 position of guanine. Temozolomide works better when MGMT is silenced by methylation of the cytosine-phosphate-guanine site (CpG) within the promoter [7]. Because of the diversity in patient responses to the present standard of care, greater personalized medicine and individualization of treatment strategies are required. The methylation state of the promoter of the MGMT gene is an essential indicator of tumor cells' response to TMZ chemotherapy [8].

Methylation-specific polymerase chain reaction (MS-qPCR) is considered a standard method for determining the MGMT methylation status [9]. The utilization of MS-qPCR has a notable advantage in terms of cost and labor efficiency when compared to alternative approaches. Furthermore, MS-qPCR can be implemented using basic equipment [7]. MS-qPCR using TaqMan probe technology provides a convenient method for semi-quantitative assessment of methylation status, eliminating the need for time-consuming gel electrophoresis. Our objective was to conduct the initial diagnosis of a novel method in our laboratory by determining the status of MGMT promoter gene methylation in GBM.

## Methods

**Patient classification:** eight Moroccan patients with primary glioblastoma were included in this study. Clinical information such as patient age at diagnosis, sex, and brain tumor localization were obtained from hospital reports.

**Tumor sample:** after the selection of formalin-fixed and paraffin-embedded blocks (FFPE), tumor tissues were collected from eight different individuals who had a histological classification of glioblastoma. The hematin-eosin slides were re-examined by an experienced pathologist to confirm the diagnosis and to select the tumor lesion where DNA can be recovered, to make sure that it contains a high concentration of tumor cells. We prepared 3 immunohistochemistry slides for each of the 8 FFPE tissue blocks for antibody anti-IDH1(R132H), anti-p53, and anti-ATRX immunostaining. Regarding MGMT methylation we prepared 5-6 slides with 6µ-FFPE slices in each one.

**Deoxyribonucleic acid extraction:** after selection of the tumor lesion in each slide of the eight glioblastomas FFPE blocks, DNA was isolated using deparaffinization of FFPE samples, and DNA extraction was carried out using the Maxwell® 16 FFPE Tissue LEV DNA Purification Kit according to the manufacturer´s instructions, this kit has been used with the Maxwell® 16 (Promega). The quantity and quality of DNA were measured using a NanoVue™ Plus Spectrophotometer at A260/A280 and A260/A230 (Biochrome).

**Bisulfite conversion:** the EZ DNA Methylation-Lightning™ Kit was used according to the manufacturer´s instructions. Treating DNA with bisulfite chemically converts non-methylated cytosines into uracil, and methylated cytosines remain unchanged. DNA denaturation and bisulfite conversion processes were combined with heat (using an Agilent thermocycler) to facilitate rapid denaturation.

**O6-methylguanine-DNA methyltransferase methylation by MS-qPCR:** quantitative methylation-specific PCR (MS-qPCR) was used to quantitatively evaluate and calculate the methylation ratio of the MGMT promoter. Using the AriaMx Real-time PCR System (Agilent Technologies, Santa Clara, CA, USA), amplification reactions were performed on 96 well plates. The PCR Kit used was Genmark´s MGMT Methylation Analysis Kit and contained the following reagents; 2X Master Mix (Taq DNA polymerase, dNTPs, MgCl2, and reaction buffer), 4X MGMT Primer Probe Mix, positive control (PC) 100% methylated DNA, and MGMT Calibrator 2% methylated DNA (theoretical). The whole reaction volume was 20µl (10µl Master Mix (2X), 5µl MGMT Primer Probe Mix (4X) and 5µl of bisulfite converted DNA template). The PC, no template control (negative control), and the calibrator were used in every run. The PCR program included an initial denaturation at 95°C for 15 min, followed by 45 cycles at 95°C for 20 and 60°C for 1 minute. The default settings in the software were used to process and export the results (Agilent). The amplification curve provides insights into the DNA amplification process over time. For our analysis, the Ct value of the endogenous control (VIC) should fall within the range of 20 to 31 (20≤ Ct(patient) ≤ 31), ensuring accurate results. Additionally, in methylation assessment, the acceptable Ct value for methylation positivity or the methylation target channel (FAM) should not exceed 42 (Ct value ≤42). The percentage of methylation was carried out using the following formula 1,9ΔCt (patient) /2ΔCt (PC)*100*conversion factor. We calculated the conversion factor value using the following formula: calibrator methylation ratio % theoretical/calibrator methylation ratio % observable. The calibrator methylation ratio observable is calculated by using the following formula= 1.9 ΔCt (Calibrator)/2 ΔCt (PC)*100.

**Immunohistochemistry (IHC) for IDH1, ATRX and p53:** deparaffinization, rehydration, and demasking of a 4-µm slice from a typical tumor FFPE block by heat-induction (water bath) at 98°C and citrate buffer (target retrieval solution high pH(50x)) for 30 min. To block the activity of the enzyme peroxidase, the slides were treated with 3% hydrogen peroxide (H_2_O_2_) for 10 min and then rinsed with distilled water and washed with wash buffer. Following that, the slides were treated with the three primary antibodies (200µl for each slide for 30 minutes) directed against IDH1 (Mouse, Monoclonal, BIOSB, IHC132), p53 (Mouse, Monoclonal, BIOSB, DO-7) and ATRX (Mouse, Monoclonal, BIOSB, BSB108) at room temperature. The EnVision detection system based on the secondary antibody with the horseradish peroxidase (HRP) (Agilent Dako) was used to identify immunoreactions, and the slides were incubated in a solution containing the peroxidase-conjugated marker polymer (200µl for 30 min. 3.3'-Diaminobenzidine (DAB) (200µl for 10 min) was used to visualize the reactions, and hematoxylin was used as a counterstain. Finally, the samples were dehydrated with alcohol buffers at different concentrations and mounted. The interpretation of p53 was based on the percentage of stained nucleus of tumor cells, we also considered stained tumor cells positively > 10% is correlated with over-expression which is the muted form of p53 (p53-mt).

## Results

**Clinical data:** the most prevalent sites of the tumor were the frontal and temporal lobes, after the parietal lobe. Table 1 lists the clinical information of the patients.

**Table 1:** clinical information of the patients

ID samples	Sex	Age at diagnosis	type of surgery	Tumor location
S1	M	56	Partial resection	Frontal
S2	F	30	Biopsy	Temporal
S3	M	61	Partial resection	Temporal
S4	M	20	Partial resection	Parietal
S5	M	36	Partial resection	Frontal
S6	M	59	Biopsy	Frontal
S7	M	10	Partial resection	Temporal
S8	M	24	Partial resection	Parietal

**Figure 1:**
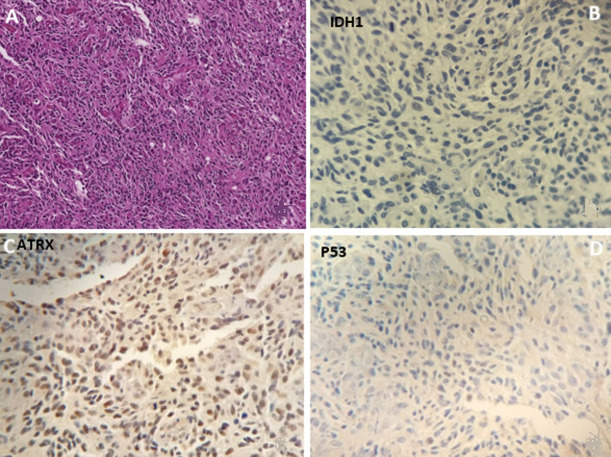
the figures depict representative images from a case study of glioblastoma (GBMs) by hematoxylin-eosin-stained tissue slide (A); and normal protein expression of isocitrate dehydrogenase 1 (IDH1-) (B); immunopositivity of alpha thalassemia/mental retardation syndrome X-linked (ATRX+) (C); and tumor protein 53 unmuted (p53-) (D); all four slides at a magnification of x10

**Immunohistochemistry:** the immunohistochemistry results showed that all glioblastomas expressed wild-type IDH1-protein, which is in agreement with the new 2021 World Health Organization Classification of Tumors of the Central Nervous that indicates the grade 4 glioblastomas do not have the mutated form of the IDH gene. Six glioblastomas in this case series had lost nuclear expression of ATRX (ATRX-), and only two of the GBMs had a nuclear overexpression of P53-protein (P53+) (Figure 1).

**Figure 2:**
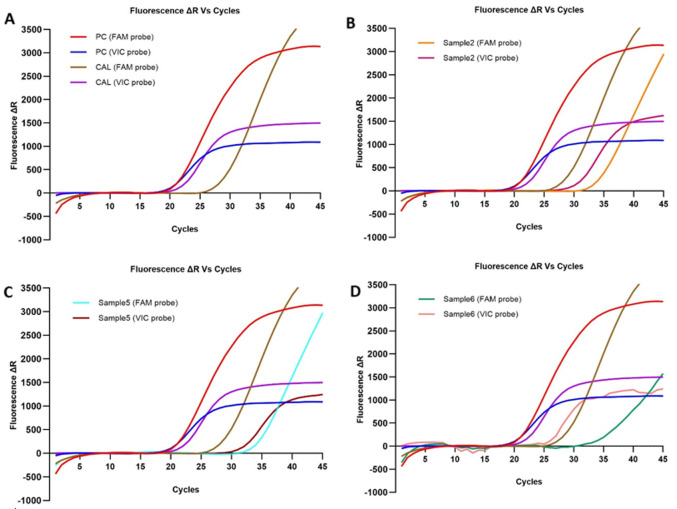
Methylation Specific qPCR (MS-qPCR) examples in the analyzed tumor samples for glioblastoma patients: (A) the standard amplification curve of positive control (PC) and calibrator (CAL); the FAM (methylated) and VIC (reference gene) probes were amplified (100% and 2%, respectively); (B, C, D) examples of methylated amplification curves for samples 2, 5, and 6 (6,8%, 27%, and 0,05% respectively)

**MGMT promoter methylation status:** real-time quantitative methylation-specific PCR is performed to determine the status of DNA methylation in 8 FFPE glioblastomas (6 partial resection FFPE and 2 biopsy FFPE). The results of the methylation analysis of the MGMT gene promoter in eight glioblastoma cases were obtained from the amplification curves and are summarized in Table 2. In the amplification curves of the methylated samples, we can observe the extent of gene target (MGMT promoter) amplification, which should be set at a value where the amplification curve slope is stable and not exponential (Figure 2), the amplification curve of the methylation MGMT primer at the PCR cycle is represented by mCt (Ct of FAM), whereas the reference gene is represented by rCt (Ct of VIC). To quantify methylation status, the ΔCt value was assessed, which signifies the variance between rCt and mCt (rCt value - mCt value). By applying the formula described in the Materials and Methods section. The qualitative and quantitative analysis of the cases studied reveals that samples 2, 5, and 6 (preliminary result for sample 6) present positive methylation of the promoter of the MGMT gene, while the other five were unmethylated. The methylation cut-off point, set at 0.6%, was determined based on the manufacturer's recommendations for the kit used in our study. Consequently, the samples were categorized as methylated if the degree of methylation exceeded the threshold of 0.6% and unmethylated if it fell below that threshold. Therefore, we considered the sample 6 as unmethylated (methylation ration = 0.05%).

**Table 2:** results of the methylation analysis of the MGMT gene promoter in eight glioblastomas

ID samples	methylome status	Methylation ratio (%)	rCt	mCt	Delta Ct
PC	**methylated**	100	17.93	21.19	-3.26
NC	**unmethylated**	0.00	0.00	0.00	0.00
CAL	**methylated**	2.00	19.19	28.22	-9.03
S1	**unmethylated**	0.00	20.67	No Ct	No ΔCt
S2	**methylated**	6.80	27.11	33.92	-6.81
S3	**unmethylated**	0.00	27.83	No Ct	No ΔCt
S4	**unmethylated**	0.00	25.48	No Ct	No ΔCt
S5	**methylated**	27.00	29.41	34.27	-4.86
S6	**methylated**	0.05	20.43	35.22	-14.79
S7	**unmethylated**	0.00	27.64	No Ct	No ΔCt
S8	**unmethylated**	0.00	27.10	No Ct	No ΔCt

rCt: Ct value of VIC, mCt: Ct value of FAM, No ΔCt= rCt value - mCt value

## Discussion

In this study, we examine the expression of IDH1, p53, and ATRX and detect the level of MGMT promoter methylation in glioblastomas. Prior research conducted by numerous authors has consistently demonstrated that patients with MGMT promoter methylation exhibit superior outcomes. This methylation status is typically assessed using Methylation Specific-PCR in routine clinical practice [7].

In this study, the methylation status was evaluated with MS-qPCR in a small series of FFPE samples, introducing the ΔCt value to quantify MGMT promoter methylation using the methylation ratio formula (Figure 2). MS-qPCR is an effective initial approach for quantifying MGMT methylation status because of its widespread accessibility, simplicity of interpretation, and low cost [8].

The technical cutoff has been demonstrated to be a reliable indicator of outcome in GBM trials involving TMZ treatment for patients [8]. Our objective was to establish this novel molecular marker in clinical practice and first use a technical cutoff to separate methylated and unmethylated patients. Furthermore, we aim in future to evaluate, optimize, and validate our cutoff point by GBM clinical trial studies. The promoter regions of the MGMT gene comprise 98 CpG sites, with only a subset of these sites utilized for the conventional MS-qPCR method [10]. The level of MGMT promoter methylation varies among tumor cells, and contamination by normal cells is common in all samples, impacting the results of real-time MS-qPCR [11].

## Conclusion

Adult gliomas are the most prevalent primary brain tumors. The most common and aggressive type of these gliomas is called glioblastomas (glioblastomas *IDH1-wt*Grade 4, 2021 World Health Organization Classification of Tumors of the Central Nervous). Despite the small number of cases in this study, it is the first in Morocco to evaluate the technical validation of MS-qPCR and demonstrate its effectiveness. We identified three cases with positive methylation among eight cases, with the observation of the expression profile of three proteins (*IDH1, ATRX and p53*) by immunohistochemistry. Our study should be expanded in large prospective studies to confirm the findings and draw definitive conclusions. This could lead to a more precise analysis of MGMT promoter methylation, potentially improving the stratification for temozolomide administration. O6-methylguanine-DNA methyltransferase gene promoter methylation should be considered as a stratification factor in future clinical trials for glioblastoma patients.

### What is known about this topic

- O6-methylguanine-DNA methyltransferase gene (MGMT) promoter methylation statute considered as a predictive and theragnostic biomarker in glioblastomas IDH-wild-type;
- The expression of mutated proteins detected by immunohistochemistry, along with the determination of MGMT promoter methylation, both contribute to predicting a better response to glioblastoma treatment;
- For assessing the MGMT methylation status, methylation-specific polymerase chain reaction (MS-qPCR) is regarded as a standard technique.

### What this study adds

- This study emphasizes the initiation of integrating MGMT gene promoter methylation into clinical practice for patients with glioblastoma;
- This study offers preliminary data to inform future research on the diagnostic and prognostic evaluation of glioblastomas in Morocco and across Africa.
